# Use of any contraceptive method among women in rural communities in the eastern region of Ghana: a cross-sectional study

**DOI:** 10.1186/s12889-023-16795-1

**Published:** 2023-10-05

**Authors:** Isaac Yeboah, Martin Wiredu Agyekum, Joshua Okyere, Ronald Osei Mensah, Mary Naana Essiaw, Hilda Appiah, Andrew Kweku Conduah, Seth Nana Kwabena Koduah, Aaron Kobina Christian

**Affiliations:** 1 Institute of Work, Employment and Society, University of Professional Studies, Accra, Ghana; 2 Institute for Educational Research and Innovation Studies (IERIS), University of Education, Winneba, Ghana; 3 Department of Population and Health, University of Cape Coast, Cape Coast, Ghana; 4 Centre for Languages and Liberal Studies, Takoradi Technical University, Takoradi, Ghana; 5 Commonwealth Senior High School, Lartebiokoshie, Accra Ghana; 6 Regional Institute for Population Studies, University of Ghana, Legon, Ghana

**Keywords:** Prevalence, Determinants, Contraceptives, Rural, Communities, Eastern, Region, Ghana

## Abstract

**Background:**

In Ghana, there is an increase in contraceptive use for traditional and modern methods in rural areas. This study seeks to examine the prevalence and determinants of current use of any contraceptive method among women of reproductive age in the rural Eastern Region of Ghana.

**Methods:**

A community-based cross-sectional study was conducted among women of reproductive age in the rural Eastern region of Ghana. A structured questionnaire was used to interview women in rural Lower Manya and Upper Manya Krobo districts of Eastern region who were selected using a simple random sampling technique. The data were analysed using Stata version 16. A Binary logistic regression was used to examine the determinants of current use of any contraceptive use (traditional and modern methods).

**Results:**

The prevalence of contraceptive use was 27.8%. In the adjusted analysis of binary logistic regression, contraceptive use was significantly lower (aOR = 0.24; 95%CI = 0.10–0.56; p = 0.001) among respondents aged 41–49 years compared to those aged 18–35 years. Contraceptive use was significantly lower among migrants (aOR:0.53; 95%CI:0.28–0.99; p = 0.048) compared with non-migrant.

**Conclusion:**

The prevalence of any contraceptive use among rural women was low. Government and other stakeholders need to create awareness about contraception in the rural areas of Eastern region of Ghana and that would help increase contraceptive methods utilization. In addition, family planning programs should target migrants to design an intervention to increase contraceptive use in rural areas.

**Table:** 

**Text box 1.** Contributions to the literature
• Research has shown that the use of any contraceptive method is increasing in rural areas of sub-Saharan Africa. For policy makers, the use of any contraceptive method even in the rural areas is heterogenous and context specific.
• Although we found that the study areas contraceptive prevalence level was similar to national rural prevalence level, the factors associated with the use of any contraceptive method in our study area provide policy makers insights into real-world.
• These findings contribute to gaps in the literature where less is known about the contraceptive behaviour of rural women in Eastern region of Ghana. Therefore, policy makers should provide contraceptive interventions targeted at migrant in rural Eastern region.

## Background

Globally, about 190 million out of 1.9 billion women within the reproductive age (15–49 years) who want to avoid pregnancy do not use any contraceptive method. It is estimated that 23.7% of women in sub-Saharan Africa who want to avoid pregnancy do not use any contraceptive method; a term referred to as “unmet needs for family planning” [1]. In Ghana, about 30% of married women have an unmet need for family planning [2]. The high unmet need is associated with the non-use of contraceptives which leads to unwanted pregnancy and consequently adverse related pregnancy outcomes such as abortion and mortality [3]. Evidence shows that contraceptives are very essential and remain a global intervention for preventing unwanted pregnancy and related maternal morbidity and mortality [4, 5]. Even though contraceptive awareness is very high in SSA, most women do not use contraceptives [6].

In sub-Sahara Africa, contraceptive use is a complex individual choice that is influenced by a variety of contextual factors including socio-demographic, cultural, economic factors and partner influence [7–17]. However, cultural, socio-demographic and economic factors tend to influence contraceptive use in rural areas in SSA [18–20]. In addition, unavailability of contraceptive services, limited knowledge among both women and their partners and perceived side effects of modern contraceptive methods could also influence the decision for women to use contraceptives most especially in rural areas [21, 22].

According to the 2017 Ghana Maternal and Health Survey, the contraceptive prevalence rate was 25% for all methods. Although there is a preponderance of empirical studies on the prevalence and determinants of contraceptive use in Ghana, most of these studies have mainly focused on modern contraceptive use [16, 23–26]. There are few studies on the determinants of contraceptive use for any method in rural Ghana [27, 28] and none of these studies is in the rural Eastern Region of Ghana to the best of our knowledge. Contraceptive prevalence (35.6%) for any method in the Eastern region is high, [7] but the associated factors, especially in the rural areas, are unknown. This is because there are no studies examining the prevalence and determinants of contraceptive use for any method (that is either traditional or modern methods) in the rural Eastern Region of Ghana. Hence, it is very imperative that we understand the factors associated with contraceptive use among rural women in the Eastern region of Ghana to help policymakers to design an appropriate intervention to help increase contraceptive use. Therefore, this study seeks to examine the prevalence and factors associated with use of contraceptives among women living in rural communities in the Eastern region of Ghana. We hypothesized that the use of any contraceptive method is associated with social and demographic factors.

## Methods

### Study setting

Ghana is basically an agrarian economy with the agricultural sector made up of predominantly smallholder farmers. The agricultural sector employs about 58% of women. The study was conducted in the rural farming communities of two Manya Krobo districts (Upper Manya Krobo districts and Lower Manya Krobo district) in the Eastern Region of Ghana. The region has the highest unmet need of 35.1% in Ghana and by extension, those in the rural areas have higher unmet needs than the urban areas. The districts are in the semi-equatorial climate belt, with annual rainfall ranging from 900 to 1,500 millimetres. The majority of the population in Upper Manya Krobo reside in rural areas (69%) while about 1 out of 4 of the population (24.2%) in Lower Manya Krobo reside in rural centers [29]. Both districts have about fourteen communities.

### Study design

The study design was a cross-sectional household survey collecting information on women empowerment and some individual and household outcomes. Primary data was collected using a structured interviewer-administered questionnaire that was adopted from standard questionnaires including Pro-WEAI (Women’s Empowerment in Agriculture Index). The Pro-WEAI questionnaire covered a range of topics such as social and demographic information, role in household decision-making around production and income, access to productive capital, access to financial services, time allocation, group membership, physical mobility, reproductive history of respondents. Hence, the current analysis is part of a larger cross-sectional household survey collecting information on women’s empowerment and their nutritional outcomes (e.g., overweight and/or obesity), health and household outcomes.

### Study population and sampling and sample size

Households with women were identified by using a 2-stage sampling procedure, in which 4 primary units (communities) were selected with probability proportional to size, and secondary units (women) were selected using the random-walk method commonly used in EPI cluster surveys to identify participants [30, 31]. The sample size for this study was based on determining the prevalence of overweight/obesity among women in the Eastern region. The formula below was used to determine the sample size. 

$$n=\frac{{Z}^{2}P\left(1-P\right) }{{d}^{2}}$$

where n = Sample size, Z = Z statistic for a level of confidence: Z = 1.96 for level of confidence of 95%, P = Expected prevalence or proportion: The prevalence of overweight/obesity reported in the 2014 Ghana’s demographic and health survey was 38.5%. This figure is expected to be rising. We assumed an overweight/obesity prevalence of 50%. This gives a minimum required sample of 385. Finally, an additional 10% of the sample was added to account for missing data or non-responding respondents. Thus, the final target sample size was 435. For the main study, we interviewed women who had ever and never had sex. For the purpose of this study, the sample unit was restricted to women who were sexually active in the last 12 months preceding the survey. This led to the reduction in the sample size for this study from 435 to 281.

### Data collection procedure

The data was collected between June and July 2021. Data collection was conducted in four communities within the two districts. These communities are Sekesua, Mensah Dawa, Oborpah and Yoguse. These were communities with most agricultural activities. Training was conducted for field enumerators on data collection processes, confidentiality, study objectives and rationale of the project. After the training, the study instrument was piloted to assess its clarity and suitability. Respondents were asked to sign an informed consent form before the interviews were conducted. Random selection of households that practice agricultural activities as main occupation was used as a sampling strategy. Using an interviewer-administered questionnaire, data were collected on socio-demographic variables such as age, educational level, marital status, household wealth status, and current contraceptive use. Reproductive history: parity and number of living children, age at first pregnancy, number of pregnancies and number of child loss were also assessed.

### Ethics statement

The study’s ethical protocol was approved by the Noguchi Memorial Institute of Medical Research Institutional Review Board, University of Ghana, Legon (NMIMR IRB Number: 020/19–20). Informed consent for voluntary participation was obtained from participants before being interviewed. Additionally, the purpose of the study including the general objectives, benefits, and risks of taking part in the study was explained to the participants before they consented to be part of the study. Participation was voluntary and remunerative (participants were given soap after the interview). Confidentiality was upheld throughout the study. The study was conducted in accordance with the relevant guidelines and regulations specified by the Ethics Committee.

### Measurement of variables

The main dependent variable for this study is the current use of any contraceptive method. Respondents were asked if they were using any family planning method to prevent pregnancy. The question posed was ‘Are you currently doing something or using any method to delay or avoid getting pregnant’? Those who were using a method, either modern or traditional method to prevent pregnancy were classified as “yes” whiles those who were not using anything to prevent pregnancy were classified as “no”.

The independent variables for the study are age, marital status, educational attainment, migration status, number of pregnancies, desire for another child, number of living children, district, type of household, head of household and occupation. The age of respondents was classified as “18–35 years”, “36–40 years”, and “41–49 years”. Marital status of respondents was categorised as “currently married”, “cohabiting” and “currently not married”. Educational attainment was classified as “no education”, “primary”, “Junior High School (JHS)”, and “Senior High School (SHS) or higher”. Migration status was classified as “migrant” and “non-migrant”. Number of pregnancies was recategorized as “1–3”, “4–5” and “6 and more”. All the women interviewed in this study have ever been pregnant. Likewise, the number of living children. Therefore, there was no zero-category number of pregnancies and living children. The number of living children was classified as “1–3”, “4–5” and “6 and more”. Age at first pregnancy was categorized as “<18 years” “18–24 years” and “25 years and above”. Access to health facility was classified as “yes” and “no”. In addition, head of household was categorized as “self”, “husband” and “other relatives”. Lastly, occupation was recategorized as agricultural self-employed and other activities.

### Data analysis

The characteristics of the study sample were described using percentages and frequencies. The association between the current use of any contraceptive methods and women’s characteristics were tested using cross-tabulation and chi-square. A multivariate binary logistic regression analysis was performed to examine the factors associated with the current use of any contraceptive method. A binary logistic regression was used because the dependent variable was a dichotomous variable. The model accounts for controlled variables, hence produces an adjusted odds ratio.  The analyses were performed using Stata version 16 and statistical significance was set at the 5% alpha level (p < 0.05).

## Results

### Description of background characteristics

From Table 1, there was a marked difference in the prevalence of the current use of any contraceptive method. The results show that 27.8% of women who were sexually active and within their reproductive age were using any contraceptive method while 72.2% women were not using any contraceptive method to prevent pregnancy. Almost 40% of the women were aged 18–35 years with a little over a quarter (26.7%) and one-third (33.5%) were aged 36–40 years and 41–49 years respectively. Most of the participants were married (43.1%), have no education (29.2%) and were non-migrants (65.1%). More than over one-third (35.9%) of the women had 1–3 pregnancies. Similarly, about 7 in 10 women (76.5%) live in households with husbands as the head of the household. Majority of the respondents (80.8%) are in agriculture as their main occupation.

**Table 1:** Characteristics of participants (n = 281)

Characteristics	Category	Frequency (%)
Any Contraceptive use	Yes No	78(27.8) 203(72.2)
Age	18–35 36–40	112(39.9) 75(26.7)
	41–49	94(33.5)
Marital Status	Currently Married Cohabiting	121(43.1) 118 (42.0)
	Not currently married	42(14.9)
Educational Attainment	No education Primary JHS	82(29.2) 68 (24.2) 63 (22.4)
	SHS or Higher	68 (24.2)
Migration Status	Migrant	98(34.9)
	Non-migrant	183(65.1)
Number of Pregnancies	1–3	101(35.9)
	4–5 6 or more	99(35.2) 81(28.8)
Number of living children	1–3	134(47.7)
	4–5	112(39.9)
	6+	35(12.5)
Age at first pregnancy	< 18 years 18–24 years	45(16.0) 196 (69.8)
	25+	40 (14.2)
Access to health facility	Yes	181 (64.4)
	No	100 (35.6)
Head of Household	Self	52(18.5)
	Husband	215(76.5)
	Other Relatives	14(5.0)
Occupation	Agricultural-self employed Other	227(80.8) 54(19.2)

Source: Pro-WEAI Data, 2021

### Association between current use of any contraceptive method and background characteristics

According to Table 2, contraceptive use by those aged 18–35 years was 36.6%. Among study participants who were currently married, 29.8% were currently using any contraceptive method and 30.2.% of those who had attained JHS education were also using any contraceptive method. It was noted that 30.6% of the women who were non-migrant were currently using any contraceptive method. It was observed that current contraceptive use was 29.6%, and 28.6% and among participants with 6 or more pregnancies, and those with 4–5 living children respectively. Contraceptive use among households’ heads with husband/partners and other relative as head of the household was 31.2% and 28.6% respectively (Table 2).

**Table 2:** Prevalence of contraceptive use by background characteristics

Characteristics	Category	Contraceptive use Yes (%)	X^2^ (p value)
Age (n = 281)	18–35 36–40	36.6 29.3	10.9 (0.004)
	41–49	17.0	
Marital Status	Not married	16.7	3.0(0.220)
	Currently married	29.8	
	Cohabiting	29.7	
Educational Attainment	No education	25.6	0.5 (0.915)
	Primary	29.4	
	JHS	30.2	
	SHS or Higher	28.6	
Number of Pregnancies	1–3	28.7	0.4 (0.780)
	4–5 6 or more	25.3 29.6	
Number of living children	1–3	28.4	0.4 (0.787)
	4–5	28.6	
	6 or more	22.9	
Migration Status	Migrant	30.6	0.6(0.434)
	Non-migrant	26.3	
Head of Household	Self	13.5	6.5(0.038)
	Husband/Partner	31.2	
	Other Relative	28.6	
Occupation	Agricultural- self employed	26.9	0.4 (0.497)
	Other	31.5	
Access to health facility	Yes	27.6	0.0 (0.946)
	No	28.0	
Age at first pregnancy	< 18 years	26.7	2.6 (0.264)
	18–24 years	30.1	
	25+	17.5	

*The bivariate result is presented in a row percentage.* Source: Pro-WEAI, 2021

### Factors associated with the current use of any contraceptive method

Table 3 presents the crude and adjusted odds of the current use of contraceptives. The results show that the odds of current use of any contraceptive method were lower (aOR = 0.24; 95%CI = 0.10–0.56; p = 0.001) among those aged 41–49 years compared to those aged 18–35 years. Migrants were less likely (aOR:0.53; 95%CI:0.28–1.01; p = 0.048) to use any contraceptive method than non-migrants.

**Table 3:** Factors associated with contraceptive use among study participants

Age	Adjusted Odds Ratio	p value	95%CI	
**Age**				
18–35 (ref)				
36–40	0.52	0.099	0.24	1.13
41+	0.24	**0.001**	0.10	0.56
**Marital Status**				
Not married (ref)				
Married	1.38	0.644	0.35	5.41
Cohabiting	0.91	0.889	0.25	3.37
**Educational Status**				
No education (ref)				
Primary	0.89	0.780	0.41	1.97
JHS	0.96	0.924	0.42	2.19
SHS or Higher	0.75	0.491	0.32	1.72
**Number of living child**				
1–3 (ref)				
4–5	1.02	0.961	0.40	2.61
6 or more	0.62	0.481	0.16	2.34
**Head of Household**				
Self (ref)				
Husband/Partner	2.94	0.082	0.87	9.92
Other Relatives	2.34	0.273	0.52	10.65
**Migration Status**				
Non-migrant (ref)				
Migrant	0.53	**0.048**	0.28	0.99
**No. of Pregnancies**				
1–3 (ref)				
4–5	1.33	0.563	0.51	3.51
6 or more	2.30	0.161	0.72	7.35
**Occupation**				
Other (ref)				
Agric-self employed	0.78	0.501	0.38	1.60
**Age at first pregnancy**				
< 18 years (ref)				
18–24 years	1.35	0.456	0.61	3.01
25+	0.88	0.821	0.28	2.78
**Access to health facility**				
Yes (ref)				
No	1.17	0.614	0.64	2.14

Abbreviations: ref -reference category; CI- Confidence Interval Source: Pro-WEAI, 2021

## Discussion

This study examined the prevalence and determinants of the current use of any contraceptive method in the rural Eastern region of Ghana. The study is relevant as it highlights the prevalence and determinants of the current use of any contraceptive method in the rural Eastern region of Ghana. Previous studies on contraceptives have focused on modern contraceptives in the urban areas and the entire country with few studies in rural areas [27, 28]. However, few studies in rural areas do not incorporate contraceptives for any method. Therefore, it is very important we understand the use of contraceptives for any method in the rural Eastern region of Ghana to help inform policymakers on the need to improve contraceptive interventions.

The overall prevalence of contraceptive use was 27.8%, which is very low despite the government of Ghana’s intervention and strategies to increase the use of family planning in Ghana. Though the prevalence rate is low, it is close to the national prevalence rate (25%) reported by the Ghana Maternal and Health Survey in 2017 [7]. This difference could be attributed to low access to information about family planning and contraception use. Also, the prevalence rate in this study is higher than what has been recorded in other studies. Achana et al., [32] reported 13% contraceptive prevalence rate in the Upper East rural areas in Ghana, 16.8% was reported in rural Osun State in Nigeria [33], 13.7% in rural Zambia [34] and 20% in rural Ethiopia [35]. On the other hand, a contraceptive prevalence rate (43%) higher than what was reported in this study was recorded in Zambia [36]. The low prevalence of contraceptive use in this study compared with other studies could be a result of the timing and settings of the study. Though in this study, about 7 out of 10 women had formal education, factors such as fear of side effects of modern contraceptives, myths surrounding contraceptive use, partner opposition, the dominance of men in reproductive decision-making due to the patriarchal system and limited access to family planning products/services could hinder the use of contraceptives [8, 27, 37]. Also, most of the communities in this study were remote areas hence there were few healthcare facilities to provide family services to clients.

We found that age was significantly associated with the current use of contraceptives. The results show that women who were 41–49 years were less likely to use any contraceptive method than those who were 18–35 years. This implies that adolescents and youth were more likely to use contraceptives to prevent pregnancy than adults. The findings of the study corroborate other studies that reported that age is very significant in the use of contraceptives [35, 38, 39]. In rural areas, majority of the adolescent and youth (18–35 years) are sexually active and may understand the consequences of engaging in unprotected sex. Hence, they are likely to use contraceptives to protect themselves from unwanted pregnancies or spacing births [39]. In addition, the few women who may be single may not want to give birth out of wedlock, hence, may be serious about family planning to regulate their sexual activities. In this study, the odds of contraceptive decreases with an increase in age. Though sexual activity for women in their thirties tends to be high, most of them are concerned about giving birth and may reduce contraceptive use. Therefore, they will engage in unprotected sex to prevent unwanted pregnancy. However, in the later age groups, there is a decrease in sexual activity as age increases [38]. This, therefore, reduces contraceptives use.

Migration status was found to be significantly associated with contraceptive use in the rural areas in Eastern Ghana. The findings show that migrants are less likely to use contraceptives than non-migrants. This study, however, contradicts findings from a similar study [40] which found that women who migrate, whether from rural to urban areas, rural to rural areas or between urban centers, are more likely to use contraceptives than non-migrant rural women. In this study, the probable reason could be that women migrate alone due to economic reasons and may not have their partners around. This could reduce their sexual activity or make them sexually inactive hence not using any contraceptives to prevent pregnancy. However, there may also be a further study using qualitative method to provide in-depth knowledge as to why migrants in rural areas are less likely to use contraceptives.

## Conclusion

This study indicates that the prevalence of contraceptive use is 27.8%. The factors associated with contraceptive use in the rural Eastern region of Ghana includes adolescent and young women and non-migrants. The findings suggest the need to promote use of contraceptives through reliable supply of contraceptives. Family planning programs should target migrants to design an intervention to increase contraceptive use in rural areas.

### Limitation of study

This study has a number of limitations. First, use of contraception was dichotomous, so we were unable to determine the depth or accuracy of the use of specific contraceptive method women reported they used. Additionally, numerical problems with the data prohibited us from including all contraceptive methods in the analysis. Second, the analysis would be strengthened by the inclusion of more measures at the couple’s level beyond demographics, knowledge of and attitudes towards contraception. Finally, there are limitations due to the study design. Because of the cross-sectional nature of this study, the results should be interpreted with caution as causality assumptions cannot be made. Despite these limitations, these data provide a sample of rural women to inform the literature on factors influencing contraceptive use. Moreover, the limitations of the small sample size and lack of qualitative part are recommended for further studies.

## Data Availability

Data will be available upon request from the corresponding author.
